# Supplementary material for: Development and nutritional evaluation of pomegranate peel enriched bars
Source: PLoS One. 2025 Jan 24;20(1):e0315830. doi: 10.1371/journal.pone.0315830 (PMC11760572; doi:10.1371/journal.pone.0315830)
Supplement: Attachment — Submitted filename: PONE-D-24-32412.docx [file pone.0315830.s003.docx]

Pomegranate peel, often considered a waste product, is gaining attention for its bioactive compounds. The study used well-established methods, emphasizing the potential application of pomegranate peel as a functional food ingredient. Therefore, the study is novel and relevant. However, manuscript should be improved:

1- The study mentions sourcing pomegranate peel, jaggery, and oats from local markets. However, there’s no indication of control over the quality and variability of these raw materials, which could affect reproducibility and consistency in the results. Were the pomegranates from a single variety or region?

2- While moisture content is measured for pomegranate peel powder, it would be valuable to also assess the moisture stability of the final product (bars). High moisture content could affect shelf life, microbial growth, and texture.

3- Storage and stability, microbial load and toxicity should be studied.

4- The manuscript lacks experimental design, details, and clarifications of the methods used.

5- The statistical analysis was not clarified.

6- The washing, drying, and grinding processes of the pomegranate peel could impact its bioactive compounds. Did the drying temperature affect the phenolic content? More details on the temperature and conditions used should be incorporated in the manuscript.

7- The abstract lacks specificity in results, vague statistical analysis. The abstract did not specify the evaluated parameters.

8- Repetition in the content of the results and discussion. Discussion lacks in-depth interpretation: How do the higher fibre, phenolic, and antioxidant levels in pomegranate peel powder impact the functional and health aspects of the final product? Why is T3 (15% pomegranate peel) the most acceptable in sensory evaluation? Is there a trade-off between nutritional enhancement and sensory properties (taste or texture)?

9- findings should be integrated with the final enriched bars.

10- More detail on how the addition of pomegranate peel powder influenced the bar composition is needed.

11- more discussion and interpretation should be done on sensory evaluation.

12- The manuscript could benefit from more introductory statements along the texts.
